# Microplastics provide new microbial niches in aquatic environments

**DOI:** 10.1007/s00253-020-10704-x

**Published:** 2020-06-04

**Authors:** Yuyi Yang, Wenzhi Liu, Zulin Zhang, Hans-Peter Grossart, Geoffrey Michael Gadd

**Affiliations:** 1 Key Laboratory of Aquatic Botany and Watershed Ecology, Wuhan Botanical Garden, Chinese Academy of Sciences, Wuhan, 430074 China; 2 The James Hutton Institute, Craigiebuckler, Aberdeen, Scotland ABI5 8QH UK; 3 Department of Experimental Limnology, Leibniz Institute of Freshwater Ecology and Inland Fisheries (IGB), Alte Fischerhuette 2, 16775 Stechlin, Germany; 4 Institute of Biochemistry and Biology, Potsdam University, Maulbeerallee 2, 14469 Potsdam, Germany; 5 Geomicrobiology Group, School of Life Sciences, University of Dundee, Dundee, Scotland DD1 5EH UK; 6 State Key Laboratory of Heavy Oil Processing, State Key Laboratory of Petroleum Pollution Control, College of Science and Environment, China University of Petroleum, Beijing, 102249 China

**Keywords:** Microplastics, Biofilms, Microbial communities, Microbial diversity and function, Ecological niche

## Abstract

Microplastics in the biosphere are currently of great environmental concern because of their potential toxicity for aquatic biota and human health and association with pathogenic microbiota. Microplastics can occur in high abundance in all aquatic environments, including oceans, rivers and lakes. Recent findings have highlighted the role of microplastics as important vectors for microorganisms, which can form fully developed biofilms on this artificial substrate. Microplastics therefore provide new microbial niches in the aquatic environment, and the developing biofilms may significantly differ in microbial composition compared to natural free-living or particle-associated microbial populations in the surrounding water. In this article, we discuss the composition and ecological function of the microbial communities found in microplastic biofilms. The potential factors that influence the richness and diversity of such microbial microplastic communities are also evaluated. Microbe-microbe and microbe-substrate interactions in microplastic biofilms have been little studied and are not well understood. Multiomics tools together with morphological, physiological and biochemical analyses should be combined to provide a more comprehensive overview on the ecological role of microplastic biofilms. These new microbial niches have so far unknown consequences for microbial ecology and environmental processes in aquatic ecosystems. More knowledge is required on the microbial community composition of microplastic biofilms and their ecological functions in order to better evaluate consequences for the environment and animal health, including humans, especially since the worldwide abundance of microplastics is predicted to dramatically increase.

**Table:** 

**Key Points** *• Bacteria are mainly studied in community analyses: fungi are neglected.* *• Microbial colonization of microplastics depends on substrate, location and time.* *• Community ecology is a promising approach to investigate microbial colonization.* *• Biodegradable plastics, and ecological roles of microplastic biofilms, need analysis.*

**Key Points**

*• Bacteria are mainly studied in community analyses: fungi are neglected.*

*• Microbial colonization of microplastics depends on substrate, location and time.*

*• Community ecology is a promising approach to investigate microbial colonization.*

*• Biodegradable plastics, and ecological roles of microplastic biofilms, need analysis.*

## Introduction

Plastics have been produced since the 1940s, and world production reached 360 million metric tons in 2018, which has resulted in severe plastic pollution of the environment worldwide (Verla et al. 2019). Most of the plastic wastes discharged into the environment are chemically very stable, corrosion-resistant and difficult to degrade by microorganisms, even those that are supposedly biodegradable (Lambert and Wagner 2016; Napper and Thompson 2019). Microplastics (1 μm–5 mm) in the environment can be classified as “primary” or “secondary” based on their original size (Germanov et al. 2018). However, many primary microplastics are directly produced and used in, e.g. personal care products such as toothpaste and certain cosmetics, and this provides a direct source of microplastic pollution (Zhang et al. 2018). Secondary microplastics are derived from the breaking up of macroplastics (> 2.5 cm) or mesoplastics (5 mm–2.5 cm) through various abiotic factors such as sunlight (photodegradation), weathering (mechanical breakup), erosion and aquatic immersion (Akdogan and Guven 2019; Cole et al. 2011; Ganesh et al. 2020; Law 2017; Sharma and Chatterjee 2017; Worm et al. 2017). White polyethylene pellets comprised most of the primary microplastics on beaches in the Caribbean, while only 23.1–34.3% of the total microplastics were secondary microplastics (Acosta-Coley et al. 2019). However, secondary microplastics were the main microplastic (66–88%) in downstream effluents from wastewater treatment plants (Estahbanati and Fahrenfeld 2016). Moreover, secondary microplastics in the environment seem to be composed of microplastic waste with little or no associated primary microplastic, e.g. in the south-eastern coastline of South Africa (Nel and Froneman 2015) and Japanese river environments (Kataoka et al. 2019). Regardless of the source, microplastics are now present in almost all environments worldwide. They are accumulating at increasing speed in aquatic environments, such as lakes and rivers, which act as “plastics collectors” from their terrestrial surroundings/watershed (Koelmans et al. 2019; Zhang et al. 2018). This could pose a potential threat to humans via ingestion of contaminated fish and seafood (Hale et al. 2020; Skåre et al. 2019). The behaviour and fate of microplastics in freshwater, estuarine, marine and terrestrial environments are therefore receiving extensive study (Akdogan and Guven 2019; Amaral-Zettler et al. 2015; Andrady 2011; Burns and Boxall 2018; Galloway et al. 2017; Horton et al. 2017; Koelmans et al. 2019; Oberbeckmann et al. 2015; Sharma and Chatterjee 2017; Zhang et al. 2018).

Microplastics do not solely represent inert surfaces in the often nutrient-poor water body but can also adsorb nutrients and organic matter from their surroundings which can provide essential substrates for microbial biofilm formation on the synthetic particulates (Oberbeckmann et al. 2015; Shen et al. 2019). Microplastic biofilms can therefore be regarded as a new microbial niche in the environment, particularly in pelagic waters (Arias-Andres et al. 2018a; Arias-Andres et al. 2019; Dussud et al. 2018; Frere et al. 2018; Galloway et al. 2017; Kettner et al. 2019; Kettner et al. 2017). Oberbeckmann et al. (2015) produced a comprehensive review of microbial community composition on marine microplastics, especially regarding heterotrophic bacteria. Some of the physical interactions of early colonizing microorganisms with plastic surfaces and their potential ecological effects have been summarized by Rummel et al. (2017). Possible ecological consequences of microplastic biofilm formation, subsequent increases in horizontal gene transfer among aquatic bacteria and effects on carbon cycling by microbes attached to microplastics have been detailed by Arias-Andres et al. (2019). “Plastisphere” microbial communities have been discussed with respect to diversity and function as well as the fate of plastics in the marine environment (Amaral-Zettler et al. 2020), while key differences and commonalities of microplastic-associated biofilms and influencing factors in freshwater and marine environments have also been summarized previously (Harrison et al. 2018). In this article, we first collate the richness, diversity and composition of the microbial communities (including prokaryotes, fungi and algae) recorded on microplastics. Secondly, we summarize important factors that influence the formation of microplastic biofilms and emphasize the need for further studies on biodegradable microplastics. Thirdly, we evaluate the possible functions of microplastic-associated microbial communities presented in recent studies. The main objective is to provide a comprehensive overview on the formation of microbial biofilms on microplastics and their associated community composition, function and ecological roles in the aquatic environments. We emphasize the unique role of microplastics as a new, emerging microbial niche in pelagic environments with so far unknown consequences for ecological and biochemical processes and biogeochemical cycling in aquatic ecosystems.

## Microbial communities of microplastic biofilms

### Microbial richness and diversity

Bacterial communities of microplastic biofilms are significantly different from those on natural particles, such as wood pellets (Oberbeckmann et al. 2018), or cellulose and glass beads (Ogonowski et al. 2018). However, as yet, no consistent conclusions have been drawn when comparing the diversity and richness of microbial communities on microplastics with, e.g. natural biofilms on organic aggregates. For example, microplastic biofilms in riverine and other freshwater ecosystems were typified by a lower taxa richness, diversity and evenness of bacterial assemblages compared with water and natural seston (McCormick et al. 2016; Miao et al. 2019). This pattern also held true for aquatic fungi in the Baltic Sea salinity gradient, where polyethylene and polystyrene samples had a significantly lower OTU richness, Pielou evenness and Simpson diversity than water and wood samples (Kettner et al. 2017). A similar pattern was also found for other eukaryotic organisms on the same microplastics substrates (Kettner et al. 2019). However, another study revealed that at sampling sites far from wastewater treatment plants, OTUs, and thus the diversity and richness of bacterial communities in the microplastic biofilms, were similar to those in the surrounding water, but still lower than those of natural seston (Hoellein et al. 2017). In contrast, biofilms on mesoplastics (average size 9.3 mm) collected from the Mediterranean Sea showed a higher diversity than free-living bacterial communities and those of natural seston without any differences in Chao1 richness (Dussud et al. 2018). This finding was supported by a study on microplastic biofilms in the Bay of Brest, which revealed a higher diversity and species richness compared to free-living and particle-attached bacteria (Frere et al. 2018). These partly contradictory results do however indicate that microbial biofilms on microplastics significantly differ in composition from that of the surrounding water and of biofilms on natural surfaces, e.g. organic matter aggregates and seston. The results also imply that microbial diversity and richness may greatly depend on environmental factors and the specific habitat studied.

### Microbial community composition

Bacterial community composition represents the main research target of studies on microbial communities of microplastic biofilms, and *Proteobacteria*, *Bacteroidetes* and *Firmicutes* are usually the main phyla detected (Delacuvellerie et al. 2019; Dussud et al. 2018; Frere et al. 2018; Gong et al. 2019; Jiang et al. 2018; Kirstein et al. 2018; Zettler et al. 2013). Of the species present, microbial pathogens are gaining increasing attention since certain microplastic biofilms have been shown to exhibit selective enrichment of certain bacterial pathogens (Gong et al. 2019; McCormick et al. 2016; Rummel et al. 2017; Wu et al. 2019). For example, *Vibrio* spp. were more abundant in microplastic biofilms than in natural seston (Frere et al. 2018; Kesy et al. 2019), while common human intestinal pathogens (e.g. *Arcobacter* spp.) were also enriched in microplastic biofilms (McCormick et al. 2016). Plant pathogens, e.g. *Agrobacterium* spp*.*; nosocomial pathogens, e.g. *Chryseobacterium* spp.; and fish pathogens, e.g. *Flavobacterium* spp. were found to be abundant in low-density polyethylene microplastic biofilms (Gong et al. 2019). In addition, two opportunistic human pathogens (*Pseudomonas monteilii* and *Pseudomonas mendocina*) and one plant pathogen (*Pseudomonas syringae*) were exclusively found in microplastic biofilms (Wu et al. 2019). Although often overlooked, fungal and other eukaryotic pathogens can be enriched (Kettner et al. 2017, 2019), indicating the potential of microplastics to select for and enrich both pathogenic prokaryotic and eukaryotic microorganisms.

Archaea may also be a component of microbial communities in microplastic biofilms, although archaea were not found in microplastic and mesoplastic biofilms from the North Atlantic garbage patch (Debroas et al. 2017). However, archaea of the *Crenarchaeota* group were present in all mesoplastic biofilms collected from the deep ocean (Woodall et al. 2018). In one study, pennate diatoms and *Bacillus* spp. were the most abundant members of the microbial community on marine microplastics, followed by coccoid bacteria, centric diatoms and dinoflagellates (Carson et al. 2013). In contrast, another study found that cyanobacteria were the main photoautotrophic microorganisms in marine plastics biofilms (Oberbeckmann et al. 2014), and these organisms were also particularly enriched in plastics biofilms collected from the Mediterranean Sea (Dussud et al. 2018). Stramenopiles dominated eukaryotic microorganisms on polystyrene and polyethylene terephthalate biofilms, and Viridiplantaea and Stramenopiles were the main eukaryotic taxa on polyethylene biofilms (Debroas et al. 2017). Ascomycota and Basidiomycota were the main fungal groups on plastic debris from the North Sea and Baltic Sea (De Tender et al. 2017). Fungal filaments and spores were also present on microplastic biofilms formed in sediments of the Vitória Bay estuarine system (Neto et al. 2019). Such studies indicate that microplastic biofilms offer a unique and novel niche for aquatic microorganisms with potential consequences for aquatic food webs, biogeochemical processes and animal and plant pathogenicity.

### Ecological functions of microbial communities

Whereas studies on the microbial community composition of microplastic biofilms are numerous, little is known about their ecological functions. To date, few studies have focused on the functions of microbial biofilms on microplastics (e.g. Arias-Andres et al. 2018a). One of the most studied potential functions of microbial communities on microplastics is degradation of the plastic polymers (see, e.g. Jacquin et al. 2019; Roager and Sonnenschein 2019). It has been proposed that *Alteromonadaceae* and *Burkholderiales* in poly(3-hydroxybutyrate-co-3-hydroxyhexanoate (PHBH) biofilms represent the major groups of bacteria capable of degrading PHBH (Morohoshi et al. 2018a; Morohoshi et al. 2018b). In addition, *Erythrobacter* spp. in microplastic biofilms were demonstrated to also degrade hydrocarbons (Curren and Leong 2019), while *Alcanivorax borkumensis* growing in microplastic biofilms seemed to play a key role in low-density polyethylene degradation (Delacuvellerie et al. 2019). Metabolic pathway analysis has indicated that microorganisms embedded in microplastic biofilms have lower “cell motility”, but greater “xenobiotic biodegradation and metabolism” potential (Jiang et al. 2018). Similar conclusions were also reached for plastics biofilms collected from the North Atlantic garbage patch which showed an increased potential for xenobiotic degradation (Debroas et al. 2017). Furthermore, the metabolism of amino acids, cofactors and vitamins was enhanced on microplastic biofilms (Miao et al. 2019). However, these functions were largely derived from phylogenetic analyses of microbial community composition and not to the respective transcriptomes, proteomes or metabolomes. Hence, the application of novel multiomics approaches, including metagenomics, metatranscriptomics and metaproteomics, should be integrated and used to identify likely specific metabolic functions and activities of microplastics-associated microorganisms in relation to their community composition. A knowledge of the factors controlling microbial community composition on microplastics would therefore enable better predictions of metabolic function and hence the potential ecological role of microorganisms thriving on microplastics.

From an ecological point of view, microplastic biofilms are formed by the aggregation of multiple microorganisms, and the biofilm mode of growth is generally thought to confer enhanced resistance to adverse environmental variables such as UV irradiation, heat and drying or toxic metals (Rao 2010; Schug et al. 2014; Timoner et al. 2012; Wang et al. 2020). In fact, the presence of antibiotic and metal resistance genes in microplastic biofilms was found to be in higher abundance than for the surrounding water, indicating that the microplastics could provide a repository for antibiotic- and toxic metal–resistant microorganisms (Yang et al. 2019). By using the Kirby-Bauer disk diffusion susceptibility test, Laganà et al. (2019) confirmed that various bacterial isolates from macroplastics indeed showed multiple antibiotic resistances against cephalosporins, quinolones and beta-lactams. The high potential of bacterial antibiotic resistance on plastics could also be related to increased resistance to cold or heat stress (Cruz-Loya et al. 2019) and light irradiation (Chen et al. 2019b), meaning that bacteria on plastics biofilms appear highly adaptable to a variety of environmental stresses. A recent study showed that microplastic biofilms also have the potential to increase the frequency of horizontal gene transfer, e.g. antibiotic resistance genes (Arias-Andres et al. 2018b). Thus, microplastic biofilms may act as foci for co-selection and transfer of metal and antibiotic resistant genes (Imran et al. 2019), which clearly confer survival advantages (de la Fuente-Núñez et al. 2013; Skåre et al. 2019). However, potential functional consequences have not yet been fully evaluated which is necessary to better understand the interactions between microbial communities and environmental factors that underpin the formation and stability of microplastic biofilms.

The spatial distribution of microorganisms in microplastic biofilms, as in other biofilms, is generally not random or homogeneous. Heterogeneous bacterial communities were observed around phytoplankton and bryozoan structures, which may be due to associations arising from the preferential attraction of bacteria to exuded organic matter and other nutrients by phytoplankton and hydrodynamic effects caused by the shape and structure of surface layers (Schlundt et al. 2019). Exopolysaccharides were found to play an important role in the formation of hetero-aggregates between microalgae and microplastics (Lagarde et al. 2016). However, microbe-microbe and microbe-substratum interactions in microplastic biofilms and their environmental significance remain largely unknown (Amaral-Zettler et al. 2020; Schlundt et al. 2019). Correlative network analyses based on 16S rDNA amplicon and metagenomic data indicated that key bacterial genera (e.g. *Rhodobacterales*, *Sphingomonadales* and *Rhizobiales*) represented important microbial associations within microbial communities of the plastisphere (Jiang et al. 2018). Further, both prokaryotic and eukaryotic microorganisms present within microplastic biofilms interact with each other with some forming hubs for subsequent microbial colonization (Kettner et al. 2019). Thus, microbial interactions in the plastisphere clearly have potential to affect biogeochemical cycles and food web dynamics in aquatic ecosystems. There is therefore a growing need to better examine microbial functions and interactions in microplastic biofilms in often contrasting aquatic habitats.

## Factors influencing the formation of microplastic biofilms

**Fig. 1:**
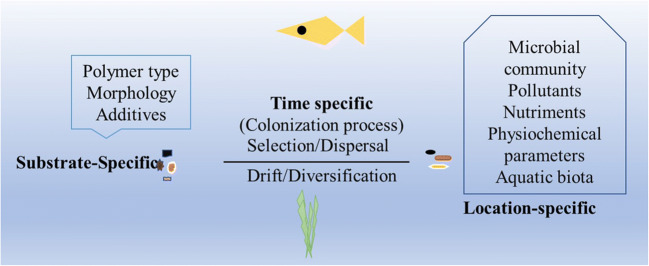
Factors influencing the formation of microbial biofilms on microplastics

The formation of microplastic biofilms includes microbial colonization of and interactions between microorganisms and microplastic surfaces under various environmental conditions. Factors influencing this process in aquatic environments can be placed in different categories: (i) microplastic characteristics (“substrate-specific”), (ii) period/succession (“time-specific”), (iii) microbial community and (iv) environmental conditions (Fig. 1). The last two factors are also referred to as “location-specific” factors (Amaral-Zettler et al. 2015; Kirstein et al. 2018; Oberbeckmann et al. 2015). Microplastic characteristics include (a) polymer type (e.g. polyethylene, polystyrene), (b) morphology (size, colour, shape, roughness, virgin or weathered) and (c) plastics additives.

### Microplastic characteristics

Polymer type is the most frequently investigated factor among all the microplastics characteristics because it directly affects microplastic biofilm formation. For example, in the Bay of Brest, microbial community composition on polyethylene and polypropylene was significantly distinct from that on polystyrene at a local scale (Frere et al. 2018; Parrish and Fahrenfeld 2019) and in the ocean on a global scale (Amaral-Zettler et al. 2015). Microbial biofilms on polyethylene terephthalate, polyethylene and polystyrene mesoplastics were dominated by *Alphaproteobacteria* and *Gammaproteobacteria*, while *Burkholderiales* (formerly *Betaproteobacteria*) dominated on polyethylene microplastic biofilms in the North Atlantic garbage patch (Debroas et al. 2017). Most studies, however, have focused on the influence of traditional non-degradable types of plastics on biofilm formation in aquatic environments (Akdogan and Guven 2019; Koelmans et al. 2019). A recent study revealed that the microbial communities of biodegradable poly(lactic acid) (PLA) biofilms were significantly different from those on seven other traditional, non-degradable plastic polymers (polyethylene terephthalate, polystyrene, etc.) (Kirstein et al. 2018). Similar results were also obtained regarding community composition on biodegradable polyhydroxyalkanoate (PHA), which were dominated by sulphate-reducing bacteria and indistinguishable in comparison to a ceramic-located biofilm (Pinnell and Turner 2019). Furthermore, degradable microplastics (e.g. PLA) can also occur in the effluents from wastewater treatment plants (Mintenig et al. 2017), but seem to be quite recalcitrant in the natural, usually nutrient-poor aquatic environment (Lambert and Wagner 2016; Napper and Thompson 2019). As well as this, bio-based and biodegradable PLA plastics may produce more microplastics during degradation compared to polystyrene (Lambert and Wagner 2016). It also should be noted that bio-based plastics and biodegradable plastics are not the same, although they are sometimes mistakenly used interchangeably. Bio-based plastics are derived from non-petroleum biological resources. Biodegradable plastics degrade via exposure to naturally occurring microbes and may be bio-based or made from petroleum (Lambert and Wagner 2017; Wackett 2019). Hence, effects of so-called biodegradable microplastics on microbial biofilm formation should be considered to better understand the fate, potential toxicity and other effects of biodegradable plastic polymers in the aquatic environment.

The roughness and hydrophobicity of microplastics constitute the most prominent parameters controlling microplastic surface properties and hence can greatly influence microbial community structure (Gong et al. 2019; Mercier et al. 2017). Aged-microplastics, produced via exposure to UV light or incubation in water for several weeks, usually have increased surface area, roughness and polarity compared to virgin samples (Brennecke et al. 2016; Jemec Kokalj et al. 2019; Liu et al. 2019; Liu et al. 2020). Such structural changes must clearly influence the formation and fate of associated microbial communities (Gong et al. 2019). Aged-microplastics, which represent the dominant microplastic type in the environment, might pose a greater threat to the aquatic ecosystem due to their high sorption capacity for hydrophobic organic pollutants and subsequent ingestion by the biota (Fu et al. 2019; Liu et al. 2019; Liu et al. 2020). Consequently, microbial community structure and function on aged-microplastics requires detailed evaluation. Effects of microplastics size on microbial community composition, however, have not been observed (Frere et al. 2018; Parrish and Fahrenfeld 2019), and there were no differences in bacterial community composition between microplastic and mesoplastic biofilms in the North Pacific Gyre (Bryant et al. 2016). In addition to size, no obvious effects of plastic shape (monofilament, sheet, etc.) on bacterial community composition were detected (De Tender et al. 2015). In contrast, additives such as plasticizers, flame retardants, pigments, antimicrobial agents and heat stabilizers, added during the production process, can determine and alter specific plastics properties (Smith et al. 2018). For example, certain pigments in plastic particles could explain differences in bacterial colonization (De Tender et al. 2015) but most effects of plastics additives on microbial community structure remain unexplored and little understood.

### Temporal succession of microbial communities associated with microplastics

Microbial community growth on microplastic biofilms represents a temporal succession process which can be divided into early, mid and late colonization periods. For example, members of the *Gammaproteobacteria* group, e.g. *Oleibacter* spp., comprised the dominant pioneer community on plastic biofilms, which were then quickly replaced by members of the *Alphaproteobacteria* and *Flavobacteria* (Pollet et al. 2018). In general, early pioneer communities in marine and estuarine microplastic biofilms belong to members of the *Gammaproteobacteria* and *Alphaproteobacteria* (Lee et al. 2008; Oberbeckmann et al. 2015). In particular, *Vibrio* species represent early colonizers of polyethylene and polystyrene microplastics in the marine environment (Kesy et al. 2019). In contrast, *Flavobacteriaceae*, *Rhodobacteraceae*, *Planctomycetaceae* and *Phyllobacteriaceae* were abundant during the later stages of the microplastics colonization (Pinto et al. 2019). Although microbial biofilms on microplastics are generally characterized by a significantly different community composition compared to free-living bacteria in that environment and natural seston, their development does largely depend on the surrounding microbial communities (Arias-Andres et al. 2018a). Microbial communities in aquatic environments such as rivers (Liu et al. 2018) and lakes (Kavazos et al. 2018) show clear geographical and depth-dependent distribution patterns which can influence the formation of specific microbial biofilms on microplastics. Thus, it is not surprising that microbial community composition of biofilms on microplastics in natural environments also depends on the microorganisms discharged into aquatic ecosystems from various sources such as wastewater treatment plants (Jiang et al. 2018). More knowledge is therefore required on the environmental factors that control microbial community structure and their related functions in microplastic biofilms.

### Environmental conditions

Environmental conditions including nutrient availability (organic/inorganic carbon, nitrate, phosphorus, etc.) for microbial growth, pollutants (toxic metals, antibiotics, persistent organic pollutants, etc.), physicochemical parameters (dissolved oxygen, light, pH, temperature, salinity, ionic strength, etc.) and aquatic biota (plants and animals) are critical factors controlling microbial biofilm formation and succession on microplastics. In lake water, temperature, nutrient levels and suspended particle concentrations determined microbial assemblages on various plastics (Chen et al. 2019a). One of the few studies on microbial functions (Arias-Andres et al. 2018a) revealed that microplastic biofilms formed in an oligo-mesotrophic lake had a higher functional richness compared to the ambient water. However, this phenomenon was not seen in dystrophic and eutrophic lakes emphasizing a strong dependency of microbial diversity and function on environmental conditions, in turn influencing microplastic biofilm formation and structure (Oberbeckmann et al. 2018). Nutrients (total nitrogen and total phosphorus) as well as salinity also influenced the growth of microbial biofilms on plastics (Li et al. 2019). Salinity seemed to be the major factor affecting bacterial diversity of plastics biofilms in an estuary (Li et al. 2019) and of microplastic biofilms in the Baltic Sea (Kesy et al. 2019). Salinity also correlated well with the abundance of potentially pathogenic *Vibrio* species (Kesy et al. 2019; Li et al. 2019), indicating a possible important connection with water hygiene and health. Since microplastic-specific bacterial communities usually encounter low nutrient levels and increasing salinities in the ocean (Oberbeckmann et al. 2018), it is not surprising that limiting carbon resources have the potential to result in specific bacterial communities tightly attached to the microplastic substrate (Kirstein et al. 2019). These results indicate that factors such as trophic mechanisms, pH and salinity represent important environmental drivers leading to specific microbial communities in various aquatic environments. The ecological and biogeochemical consequences of this process remain largely unknown due to our still limited knowledge on the linkages between microbial community composition and function.

Aquatic plants and animals play an important role in the transfer of microplastics across complex food webs (Au et al. 2017). Interactions between the microbiome of aquatic animals or of leaves and the rhizosphere of aquatic plants potentially influence microbial biofilm formation on microplastics (Jemec Kokalj et al. 2019; Rezania et al. 2018). It has also been shown that microplastics that pass through the gut of the blue mussel *Mytilus edulis* developed a similar bacterial community composition as the mussel’s gut microbiome (Kesy et al. 2017). This indicated that surrounding environmental conditions (abiotic or biotic) can shape the plastisphere microbiome to a large extent (Kettner et al. 2017) with potential consequences for ecosystem functioning.

### Development of microbial communities in microplastic biofilms

The formation and development of the microbial community structure of microplastic biofilms to a large extend depend on “location-specific”, “time-specific” and “substrate-specific” characteristics. However, most studies reveal that location-specific characteristics play a more important role than substrate-specific” factors in shaping the bacterial community composition of microplastic biofilms (Amaral-Zettler et al. 2015; Curren and Leong 2019; Kesy et al. 2019; Oberbeckmann et al. 2015). To date, little is known about the mechanisms leading to the formation and maturation of microbial biofilms on microplastics. In particular, most studies lack a conceptual framework, e.g. based on community ecology theory. This is surprising since dispersal, selection, ecological drift and diversification have been identified as the main assembly processes for microbial communities in the environment. Stochastic and deterministic processes involved in microbial selection, dispersal, diversification and drift provide a theoretical framework to better understand spatial and temporal community dynamics (Nemergut et al. 2013; Stegen et al. 2012; Zhou and Ning 2017). So far, the relative importance of stochastic and deterministic processes in shaping the microbial community structure of microplastic biofilms has been little studied (Amaral-Zettler et al. 2015) and may require new concepts and approaches to better understand plastic-specific process dynamics. This knowledge is urgently required for understanding and predicting microbial colonization on plastics and their potential ecological influence in a rapidly changing world due to global climate change and other anthropogenic impacts.

## Conclusions and recommendations

Factors influencing microbial community composition and function require further studies to better understand underlying processes and mechanisms. Most prior studies have been restricted to traditional non-degradable plastics, but the worldwide increasing use of degradable plastics means that these less refractory polymeric compounds also need to be carefully considered. A total of 127 countries have adopted some form of legislation to regulate the use of plastic bags (UNEP 2018), but most plastic pollution remains unsolved. Already in 2015, the US Microbead-Free Waters Act was established as a bipartisan agreement to eliminate preventable microplastic sources in the USA (McDevitt et al. 2017). However, the bill has been criticized for being too limited in scope and also for discouraging the development of biodegradable alternatives that ultimately are needed to solve the bigger issue of plastics in the environment. In January 2018, the European Union (EU) has released a more holistic strategy for a new plastics economy in a circular mode to reach a more sustainable plastics industry by the year 2030 (European Commission 2018). This strategy also discusses opportunities and risks of (bio)degradable plastics (European Commission 2018). More recently, the Chinese government announced a new strategy for further strengthening the control of plastic pollution in the environment (NDRC 2020). This strategy limits the use of non-degradable plastics for bags, disposable tableware and packing and promotes the use of more degradable plastics. Biodegradable plastics should therefore be the future direction and manufactured from renewable resources to allow for a circular economy, i.e. that same extent of production and recycling to reduce microplastics generation in the environment. The expected increase in “biodegradable” plastics and the dynamics of associated microbial biofilms therefore require increasing future attention from the scientific community.

**Fig. 2:**
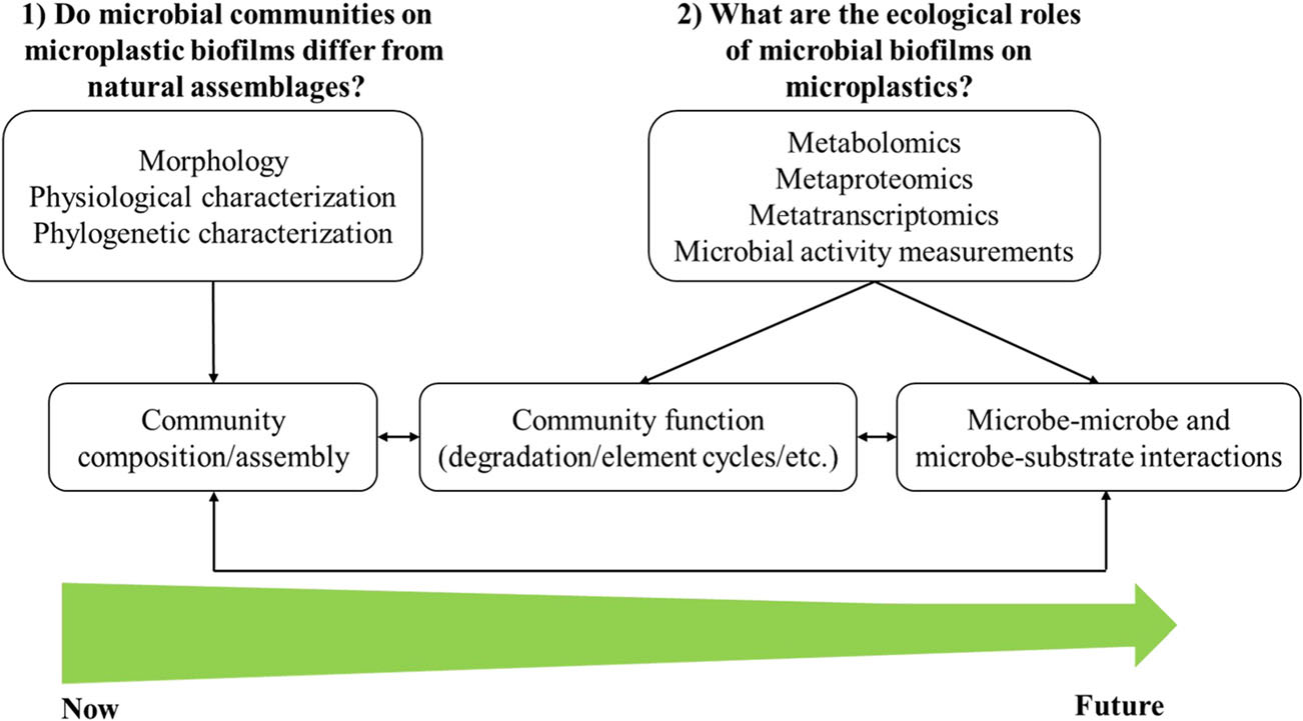
Basic questions and methodological approaches to unravel microbial community composition and their ecological roles on microplastic biofilms

The advantages and limitations of current research methods for microplastic biofilms have been summarized (see Arias-Andres et al. 2019). To date, the microbial community composition of microplastic biofilms has mainly been analysed using Illumina amplicon sequencing, and only a few studies have used shotgun metagenome sequencing. Recent improvements include analysis of full-length bacterial 16S rRNA and/or long reads of the fungal LSU, SSU and ITS gene regions based on third-generation sequencing, which has been found to effectively reduce previous methodological bias. Such improved methods allow a much higher phylogenetic resolution, often to the species level, which is essential to reliably identify potential pathogenic organisms which can be detrimental to humans and animal health. Further, shotgun metagenome sequencing in combination with metagenome-assembled genome (MAG) analyses would allow for a deeper insight into microbial diversity, evolution and potential functions of microplastic biofilms. eDNA metabarcoding can also be used to simultaneously understand community composition and the biodiversity of archaea, bacteria, fungi and other eukaryotic microorganisms in microplastic biofilms. Such approaches will allow better determination of possible microbial interactions and the key species present on various microplastic biofilms. In addition to sequencing, morphological and physiological characteristics (e.g. production of extracellular polymeric substances) should be collated to increase insights into the morphology, composition, evolution and functions of microplastic microbial communities. The combination of these methods with theories of community ecology will enable better evaluation of processes and the underlying mechanisms of microbial community dynamics in microplastic biofilms (Fig. 2).

The focus of future studies on microbial biofilms on microplastics should concentrate on functional and ecological aspects affecting aquatic food web dynamics and biogeochemical processes. Meta-transcriptomics, metaproteomics and metabolomics are now well established and important tools to assess functions and ecological roles of microbial communities (Fig. 2). These methods when combined with direct measurements of biochemical activities of microplastic biofilms, e.g. carbon assimilation and nitrogen fixation, provide a promising approach to uncover roles of microplastic biofilms in aquatic biogeochemical processes. These also include microbe-microbe and microbe-substrate interactions which, so far, have gained little attention. The wealth of current methods and approaches in environmental microbiology, geomicrobiology and microbial ecology together with relevant conceptual frameworks, based on community ecology, should provide deeper understanding of the largely understudied functions and ecological implications of microbial communities on the steadily increasing aquatic load of microplastics. Such knowledge is also required to inform future management strategies to secure water hygiene and health in times of dramatic environmental changes.
